# Barriers and Facilitators to Engaging in Smoking Cessation Support Among Lung Screening Participants

**DOI:** 10.1093/ntr/ntad245

**Published:** 2023-12-10

**Authors:** Pamela Smith, Harriet Quinn-Scoggins, Rachael L Murray, Grace McCutchan, Annmarie Nelson, Graham Moore, Matthew Callister, Hoang Tong, Kate Brain

**Affiliations:** Division of Population Medicine, Cardiff University, Heath Park, Cardiff, UK; Division of Population Medicine, Cardiff University, Heath Park, Cardiff, UK; Division of Epidemiology and Public Health Clinical Sciences, Nottingham City Hospital, University of Nottingham, Nottingham, UK; Division of Population Medicine, Cardiff University, Heath Park, Cardiff, UK; Marie Curie Research Centre, Cardiff University, Cardiff, UK; School of Social Sciences, Cardiff University, 1-3 Museum Place, Cardiff, UK; Department of Respiratory Medicine, Leeds Teaching Hospitals NHS Trust, Leeds, UK; Institute of Health Sciences, University of Leeds, Leeds, UK; Division of Population Medicine, Cardiff University, Heath Park, Cardiff, UK; Division of Population Medicine, Cardiff University, Heath Park, Cardiff, UK

## Abstract

**Introduction:**

Embedded smoking cessation support within lung cancer screening is recommended in the United Kingdom; however, little is known about why individuals decline smoking cessation support in this setting. This study identified psychosocial factors that influence smoking cessation and quit motivation among those who declined support for quitting smoking alongside lung cancer screening.

**Aims and Methods:**

Qualitative interviews were conducted between August 2019 and April 2021 with 30 adults with a smoking history, recruited from the Yorkshire Lung Screening Trial. Participants had declined smoking cessation support. Verbatim interview transcripts were thematically analyzed.

**Results:**

Fifty percent of participants were male and the majority were from the most deprived groups. Participants reported low motivation and a variety of barriers to stopping smoking. Participants described modifiable behavioral factors that influenced their quit motivation including self-efficacy, perceived effectiveness of stop-smoking services including smoking cessation aids, risk-minimizing beliefs, lack of social support, absence of positive influences on smoking, and beliefs about smoking/smoking cessation. Broader contextual factors included social isolation and stigma, coronavirus disease 2019, and comorbid mental and physical health conditions that deterred smoking cessation.

**Conclusions:**

To encourage engagement in smoking cessation support during lung cancer screening, interventions should seek to encourage positive beliefs about the effectiveness of smoking cessation aids and increase confidence in quitting as part of supportive, person-centered care. Interventions should also acknowledge the wider social determinants of health among the lung screening-eligible population.

**Implications:**

This study provides an in-depth understanding of the beliefs surrounding smoking and smoking cessation and further potential psychosocial factors that influence those attending lung cancer screening. Many of the barriers to smoking cessation found in the present study are similar to those outside of a lung screening setting however this work offers an understanding of potential facilitators that should be considered in future lung screening programs.

## Introduction

Over 85% of cases of lung cancer are caused by smoking tobacco,^1^ and research has demonstrated that stopping smoking at any age can significantly reduce lung cancer risk.^2,3^ Smoking is a major health inequality concern due to higher smoking rates among people from low socioeconomic (SE) groups in developed countries. High prevalence of tobacco smoking among low SE groups demonstrates a striking relationship between social context and health behavior.^4,5^

Lung cancer screening (LCS) using low-dose computed tomography has been implemented in nine high- and upper-middle-income countries^6^ for high-risk groups based on age and smoking history.^7^ LCS has been recommended by the UK National Screening Committee for national implementation based on the Targeted Lung Health Check model,^8^ including integrated smoking cessation service provision.^9–11^ Attendance at LCS may offer eligible individuals a “teachable moment” for smoking cessation, occurring at a time when those who smoke might be particularly receptive to offers of assistance to quit smoking.^12–14^

Previous research conducted with individuals who have a smoking history and are from a low SE background has shown that there are multiple complex barriers that may influence motivation to stop smoking including high levels of nicotine dependence, lack of confidence for quitting, low perceived effectiveness of Stop Smoking Services (SSS), risk-minimizing beliefs related to smoking-related diseases, and pre-existing physical and mental health issues.^15–19^ A further factor that may hinder smoking cessation and motivation to stop-smoking attempts in the high-risk, lung screening-eligible population is a lack of social support. People who smoke from lower SE groups report negative experiences when trying to quit, including a lack of support during previous quit attempts.^20,21^ In this population, difficult living conditions, a pro-smoking social context, and isolation from wider social norms can undermine cessation.^22–24^

The Yorkshire Lung Screening Trial (YLST) is assessing the impact of low-dose computed tomography screening on lung cancer outcomes at a population level in the United Kingdom.^25^ The YLST aims to test low-dose computed tomography screening in targeted community settings, concentrating specifically on deprived areas. Unless they explicitly decline, YLST participants are offered consultation with a specialist smoking cessation practitioner (SCP) as part of a nested substudy: the Yorkshire Enhanced Stop Smoking (YESS) study.^26^ The YESS study is testing whether the provision of enhanced, personalized information delivered by a SCP can increase cessation rates within the context of a lung screening program.^26,27^ The YESS study methods and package of smoking cessation support have previously been outlined in the study protocol.^26^

To date, no research study has examined influences on declining smoking cessation support within LCS. This qualitative interview study aimed to understand in-depth the psychosocial influences on smoking cessation and quit motivation in LCS participants who opted out of smoking cessation support in the YESS study.

## Materials and Methods

### Inclusion Criteria

**Figure 1.**
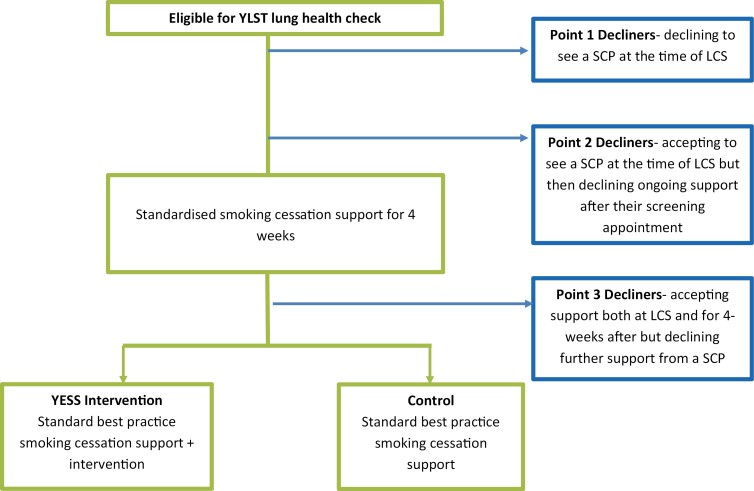
Participant recruitment flow diagram.

Participants were those who smoke, aged 55–80, who had taken part in the YLST, had declined smoking cessation support as part of the YESS study, and had consented to be contacted for a further interview. Participants could opt out of smoking cessation support at one of three time points: (1) declining to see a SCP co-located on the screening van at the time of LCS; (2) accepting a face-to-face consultation with a SCP co-located on the screening van but then declining ongoing support after their initial consultation at the screening appointment; or (3) accepting the initial face-to-face consultation on the screening van and a further 4 weeks of weekly check-in telephone-based support but declining further support from a SCP (Figure 1). These three time points took place prior to individuals enrolling into the YESS study. At 4 weeks, participants were invited to attend another face-to-face consultation where they were invited to formally join YESS (aligned to decline point 3). During the coronavirus disease 2019 (COVID-19) pandemic (from March 2020 onward), this 4-week consultation was changed to be virtual (telephone based), and all prior procedures stayed the same.

Interviews were conducted between August 2019 and April 2021. We aimed to sample participants purposively based on gender, age, and point of declining smoking cessation support. Thirteen participants were recruited before and 18 participants were recruited after the onset of the COVID-19 pandemic (March 2020). One of the recruited participants was removed at stage of analysis due to a language barrier.

### Procedure

Potential participants provided consent to be re-contacted for further research via the YLST or the YESS (depending on point of declining) and their contact details were then passed on to the YESS study team and stored on a password-protected computer. Two female qualitative researchers (PS and HQS) who were part of the study team contacted potential participants and conducted the one-to-one qualitative interviews by telephone at the researcher’s place of work and home. Prior to the interview, participants were sent a hard copy of the consent form and participant information sheet. Further consent to participate and permission to audio-record the interview were taken verbally at the point of the interview. PS and HQS also took time to build rapport, introduce themselves, and explain their role in the study. Interview duration ranged from 20 to 40 minutes. After the interview was completed, participants were sent a £10 shopping voucher as a reimbursement for their time. All audio recordings were saved onto a password-protected Cardiff University computer and uploaded to a transcription company via their secure and encrypted portal.

### Interview Schedule

A semistructured interview topic guide (Appendix S1) was developed and some of the questions were guided by the PRIME theory^28^ (eg, beliefs about smoking and plans to continue or quit smoking). Other questions, such as social networks and comorbid conditions, were identified as relevant in the field of smoking and smoking cessation for the target population.^15^ The topic guide was pilot tested on a member of the public from a low SE background who had a smoking history. Open discussions were invited on (1) delivery of stop-smoking support as part of LCS and reasons for declining stop-smoking support; (2) views on stopping smoking and their current motivation to quit using the Motivation to Stop Scale^29,30^; (3) self-efficacy in relation to quitting smoking; (4) previous quit attempts; (5) impact of their social network on smoking behaviors; and (6) any comorbid conditions that may impact their motivation to quit smoking.

Demographic measures (age, gender, postcode, and decline point) were collected from the YLST and motivation to stop smoking was captured during the interview using the Motivation to Stop Scale.^29,30^ Postcode data were used to categorize participants into deprivation deciles using the English Indices of Multiple Deprivation.^31^

### Analysis

Interviews were analyzed thematically^32^ by PS using NVivo Version 12.^33^ Both an inductive and deductive approach to analyzing the data was adopted, which involved familiarization with the data, coding and searching, reviewing, and defining emergent themes.^32^ Independent dual coding of 20% of the interview transcripts was conducted by HQS, and discrepancies were resolved through a discussion between HQS and PS who met to discuss data interpretation. Independent dual coding took place until all data had been systematically coded and transcripts were checked for the presence of any newly identified concepts.

## Results

Thirty participants were interviewed with an age range of 56–79 years. Fifteen interview participants (50%) were male. Twenty-four (80%) were residents in the most deprived 10%–50% of neighborhoods across England^34^ and the median Motivation to Stop Scale score was 2 (low motivation to stop smoking) (Table 1).

**Table 1.** Interview Participant Characteristics

	Interviewed (*n* = 30)				
	*N* (%)	Median (range)	YESS decline point 1	YESS decline point 2	YESS decline point 3
Age (years)		69 (56–79)			
Gender (%)					
Male	15 (50.0)	**—**	4 (26.7)	6 (40.0)	5 (33.3)
Female	15 (50.0)	**—**	9 (60.0)	1 (6.7)	5 (33.3)
Motivation To Stop Scale score (Median (Range))^b^		2.1 (1–7)^a^	2.2 (1–4)	1.7 (1–3)	2.8 (1–7)
YESS decline point					
Decline point 1—declining to see an SCP at the time of LCS	5 (16.7)	**—**	**—**	**—**	**—**
Decline point 2—accepting to see an SCP at the time of LCS but then declining ongoing support after their screening appointment	15 (50.0)	**—**	**—**	**—**	**—**
Decline Point 3—accepting support both at LCS and for 4 weeks after but declining further support from a SCP	10 (33.3)	**—**	**—**	**—**	**—**
Index of Multiple Deprivation decile (1 = most deprived, 10 = least deprived)	**—**	3 (1–10)	**—**	**—**	**—**
Smoking status					
Current smoker	14	**—**	**—**	**—**	**—**
Former smoker	1	**—**	**—**	**—**	**—**

^a^Motivation to Stop Scale data missing for two participants.

^b^A one-item measure with seven response categories ranging from 1 (lowest) to level 7 (highest level of motivation to stop smoking).

Five first-level themes, for which data saturation was achieved, were identified: acceptability of smoking cessation support and SCP; beliefs about smoking and smoking cessation; contextual barriers to quitting smoking; perceived effectiveness of SSS and smoking cessation aids; and social influences on smoking and smoking cessation. There were no major differences in results found between the three decline points.

### Acceptability of Smoking Cessation Support and SCP

Being approached about smoking cessation support during LCS and discussing their smoking behavior was acceptable to all participants. Participants were open to hearing from the SCP about smoking cessation with many mentioning that they expected to have a discussion about smoking at their appointment. The majority of participants felt that the SCP was approachable and considerate of their decision to continue smoking. Participants who initially accepted to have 4 weeks of telephone support from the SCP after their lung screening appointment (Decline Point 3) felt that they had built a good relationship with the SCP and felt comfortable talking to them about their current smoking behavior and past quit attempts.

*I said that I didn’t want to stop and she were very kind and said that that didn’t matter but that we could have a chat and see how things went, that she could help if I wanted (Female, 81 years, decline point 3).*

The reasons for declining support were mainly related to wider psychosocial factors that are reported below and seemed to influence participant’s motivation to stop smoking. These were unrelated to the offer of support that they experienced in the lung screening setting. For this reason, participants were unsure about what they would need during their lung screening appointment to help them stop smoking as they felt they were not motivated to do so at that moment.

*I don’t know what kind of support I’d expect (Male, 66 years, decline point 1)*

### Beliefs About Smoking and Smoking Cessation

When approached about quitting smoking during their lung screening appointment, some participants reported that discussing quitting smoking often caused them to feel anxious and therefore smoke more. Many expressed fatalistic views towards their general health and felt a general lack of control over their future health. Participants believed that the damage to their health due to smoking was irreversible and that any changes to their smoking would not have a major effect on their health.

*It was a bit awkward really, I felt that the practitioner wouldn’t help me [...] I think the more you try and convince me to stop smoking, the more I’d do it (Male, 59 years, decline point 2).*

*“It might just get a little bit better. I know it’ll never be right, I’ve done some damage” (Female, 59 years, decline point 2).*

### Smoking as a Coping Mechanism

The majority of participants felt they did not have the desire to quit smoking because smoking was the only way they knew how to cope with a variety of external circumstances. Participants described using smoking as a strategy for dealing with multiple life stressors such as financial worries, supporting unwell family members, and pre-existing health conditions, and also viewed these stressors as competing priorities that outweighed their need and desire to quit smoking.

*I now have some present family problems, I think my daughter’s inherited some mental health problems, and she’s quite a cause for concern for me really. So I think it’s [smoking] been just […] it’s been a comfort really (Male, 69 years, decline point 2).*

### Risk-Minimizing Beliefs

Some participants viewed smoking as risky but also held simultaneous views about the dangers of stopping, such as negative consequences to their health after quitting smoking.

*It’s people like the three that have given up smoking, two pass away and one’s fighting cancer, in my head I’m thinking smoking, it’s a risk as a lot of other things are but it sounds like it’s a risk if you give up after so long smoking (Female, 59 years, decline point 2).*

Participants generally compared the quantity and frequency of their smoking to other smokers. Some participants believed that they had control over their smoking behavior as they were able to limit the amount that they smoked. Participants justified their current smoking behavior by comparing it to their previous experience of smoking more cigarettes. However, some participants displayed higher levels of risk perception of smoking-related diseases and described a fatalistic attitude toward their health. For example, one participant believed that there was a high chance that they would develop cancer due to smoking and that smoking had a direct impact on the length of their life.

*And he smoked a hell of a lot more than I ever did (Male, 75 years, decline point 2).*

*It’s that fear of being a smoker how much longer have I got left. It’s not if I get cancer, it’s when I get cancer in my mind (Female, 59 years, decline point 2).*

### Self-Efficacy

Some participants lacked the confidence to initiate a quit attempt, demonstrating low self-efficacy in relation to smoking cessation. When discussing a lack of confidence in their ability to quit smoking, participants felt they did not possess the willpower to enact or sustain a quit attempt. They believed that they did not have the strength to quit smoking and did not want to make this change even if they were faced with poor health.

*I don’t try anything, I’m not trying to stop, therefore I’ve not failed, I’m just an unhappy smoker (Female, 68 years, decline point 2).*

*It goes through me mind thinking everything I say it’s all an excuse because I’m just weak and I can’t, I haven’t got the willpower to even try (Female, 59 years, decline point 2).*

### Moral Obligation to Accept Support

Participants who declined smoking cessation support after receiving four weeks of smoking cessation support (decline point 3) initially accepted the support because they did not want to let the service down. Decline point 3 participants were recruited during the COVID-19 pandemic and discussed how it felt important to them to accept the support during a time when healthcare resources were stretched.

*Well truthfully love I didn’t want to say no at the beginning. I didn’t not want to […] like I said I had no reason to say no really. So I just went along with it (Female, 81 years, decline point 3).*

*I felt bad for […] I didn’t want to say no to him (Male, 61 years, decline point 3).*

### Cutting Down as an End Goal

Some participants who declined ongoing support after 4 weeks had made changes to their smoking, having reduced the quantity smoked since attending the van and meeting the SCP. Participants were positive about the changes that they had made since meeting the SCP and believed that they had succeeded in achieving what they had set out to achieve. Participants viewed cutting down as their end goal and reflected on discussions that they had with the SCP about this behavior change.

*I don’t want to stop. I realised that with the puffer I could cut down, and I have. And that’s it for me. I’ve done what I wanted and I think I’ve gotten what I wanted to get out of it out (Female, 72 years, decline point 3).*

*It was great, really good. Wouldn’t have been able to do it without it (Male, 66 years, decline point 3).*

### Physical and Psychological Barriers

The majority of participants described experiencing comorbid physical and mental (including undiagnosed depression and anxiety) health conditions. Some participants reported having had a clinical diagnosis such as COPD and sleep apnea, and some discussed issues such as backache for which no underlying cause had been diagnosed. Participants discussed how their physical health impacted their mental health and their smoking behavior. They viewed smoking as a way to cope with physical pain and saw smoking as a form of self-medication that helped them to deal with physical issues.

*It’s the cigarettes are also one of my medications if you know what I mean. I take all my tablets and have a cigarettes and then all my problems will be all alright for a bit (Female, 59 years, decline point 2).*

Some participants described low motivation to stop smoking because of their age. Age as a barrier to quitting was demonstrated in those who felt giving up smoking was a change in their behavior that they were not willing to make at their stage of life. Participants mentioned feeling content with their life and therefore not wanting to make an attempt to stop smoking as this was not important to them.

*I don’t want to, at my age I, I really don’t want to start giving up things that, I don’t know, maybe I’m content (Male, 69 years, decline point 2).*

### Perceived Effectiveness of Stop-Smoking Services and Smoking Cessation Aids

Most participants had prior experience of unpleasant side effects from NRT while attempting to quit smoking, and these past experiences impacted their current views towards the use of NRT for quitting. There was a general lack of awareness regarding which nicotine replacement therapy (NRT) would most suit them, due to previously having bad experiences and then immediately stopping their quit attempt. Participants generally expressed negative views about e-cigarettes which were founded in conversations with friends or family members.

*But then the patches started to burn my arm [...] And I stopped using them. It just put me off a little bit (Male, 70 years, decline point 1).*

*Well I’ve got some friends what have them [e-cigarette] and they’re right fragile and plus they were charging one up once and the thing exploded (Male, 63 years, decline point 1).*

Some participants mentioned that they were not aware of local SSS available to support them and did not know how these services could help them during a quit attempt. Generally, participants were skeptical regarding behavioral counseling for smoking cessation and mentioned that they would not use this form of support in the future due to not wanting to be in a group with other people. Some participants mentioned that if they were to attempt quitting in the future, it would be a solitary pursuit and they would not utilize any support available to them.

*I don’t know anything about group ones at all really, I didn’t know they did them (Female, 68 years, decline point 2).*

*If they try and shove me off to one of them things, smoking things, I won’t go because I don’t like being round too many people, I can’t do it (Male, 63 years, decline point 1).*

*I would probably not tell anybody and do it privately to see if I could achieve it. And then it takes a little bit of the pressure of I’m failing away (Female, 68 years decline point 2).*

One participant who declined ongoing support after 4 weeks discussed how the SCPs improved their awareness of the NRT and pharmacotherapy that was available to them. Participants also felt that the conversations they had with the SCPs enabled them to understand how the NRT works and what behavioral changes they could make to encourage smoking cessation.

*The tablets were the main thing like you said […] they were the main thing that did it. But the talking with [the SCP], well without them I wouldn’t have the tablets. And knowing how to take them and what they would do and understanding how it all works. How I need to get ready for them and get rid of my cigs and things from around the house (Male, 66 years, decline point 3).*

### Social Influences on Smoking and Smoking Cessation

#### Social Isolation

Although not directly attributed by participants as a reason for declining smoking cessation support, participants discussed being socially isolated and rarely having contact with family, friends, or others within their social network. Participants spoke about how they felt lonely due to being socially isolated. Some participants also mentioned experiencing smoking-related stigma, in that those in their social circle did not want to visit them because of their smoking status which resulted in them feeling shame and worry.

*Nobody usually comes and knocks on my door, or very few people come and what have you, so I am quite lonely as well (Male, 59 years, decline point 2).*

*So people don’t come to me, because I’ve got a house that smells of smoke and it puts me into a lower depression than I’m already in (Female, 59 years, decline point 2).*

#### Social Support During a Quit Attempt

As a result of this isolation, participants lacked social support for quitting smoking and felt that they did not have anyone to hold themselves accountable to during a quit attempt. Fear of being perceived negatively through failing to quit was apparent for some participants. These individuals discussed how they would make a conscious decision not to let their family or friends know about a quit attempt in the future, in order to avoid feeling like they have failed. Additionally, one participant who declined support at point 3 reported that they would not be able to quit as their partner currently smokes in the house.

*I don’t have to answer to anybody so I could turn round and say “No I’m not doing it” (Male, 66 years, decline point 1).*

*Well my husband, he smokes all day. So if I stop it won’t make a difference. I still have the smoke (Female, 77 years, decline point 3).*

## Discussion

This research generated rich insights into the psychosocial influences on quit motivation in the context of readily accessible, community-based lung screening with integrated smoking cessation support. Findings were similar across all three points of declining smoking cessation support, with participants describing modifiable psychosocial factors that influenced their quit motivation and smoking cessation, including self-efficacy, perceived effectiveness of SSS (including NRT and behavioral counseling), risk-minimizing beliefs, social influences on smoking, and beliefs about smoking/smoking cessation. Broader contextual factors that were described included barriers such as smoking-related stigma, social isolation, COVID-19, and mental and physical health. Many of the barriers to smoking cessation found in the present study are similar to those outside of a lung screening setting. However, this work offers an understanding of potential facilitators that should be considered in future lung screening programs.

The PRIME theory states that evaluative beliefs regarding smoking can influence an individual’s motives and desires to continue/quit smoking.^28^ The results from this research demonstrate that participants experienced social isolation and a range of complex, conflicting external factors that impacted their beliefs on smoking and smoking cessation. Despite some participants reporting social isolation as a reason for continued smoking, findings demonstrated a general lack of knowledge and interest regarding in-person behavioral support from local SSS. Participants perceived SSS to be ineffective despite much evidence to the contrary.^35–37^

Lack of social connection appeared to not only foster smoking behavior, but also discourage or undermine smoking cessation. Most participants in the current study reported experiencing social isolation, in which they rarely had regular or extended contact with family or friends. Participants reflected on how this impacted their smoking behavior with many feeling they lacked the positive social support necessary for quitting smoking. Participants also reported lacking a consistent social support system and experiencing feelings of exclusion, stigmatization, and segregation, which can encourage secrecy and social withdrawal from those who do not smoke.^38^ Similarly to previous qualitative research,^39^ participants described numerous potential barriers as a result of COVID-19, including restricted access to coping strategies that were previously available such as visiting family and friends.

There was variation in risk-minimizing beliefs in the current study with some participants demonstrating low risk perception in relation to smoking-related diseases. These findings contrast previous research that has shown high perceived risk among lung screening-eligible participants who smoke.^17,40^ However, further research is needed to understand smoking-related beliefs for the target population. Those who accepted support both at LCS and for 4 weeks after, but declined further support from an SCP, also felt that smoking cessation was not a priority and they had initially accepted the support out of moral obligation. Due to unmet psychosocial needs, participants were unlikely to see quitting as a priority and considered smoking to be necessary for everyday coping and stress relief. This finding is similar to other studies that have demonstrated increased smoking as a coping strategy^41–43^ associated with having fewer materials and social resources available to effectively cope with stress, particularly for individuals who smoke from low SE backgrounds.^44–46^

Postcode-level data shows that the majority of participants recruited were from the most deprived deciles. Individual indicators of SE status were not collected and therefore caution should be made when interpreting results in relation to this study due to issues involving sample representativeness of a deprived population. However, these findings add to the limited understanding of how smoking cessation support in a lung screening context could be adapted to improve access and uptake for a lung screening-eligible population, for example raising awareness of the different forms of NRT, behavioral counseling, and the benefits of using e-cigarettes. Additionally, training SCPs in motivational interviewing techniques and embedding peer counseling for smoking cessation into community support may help to dispel stigma and fear of judgment surrounding smoking that the target population has reported experiencing.^47,48^

Acknowledging the wider social determinants of health, through adopting a “whole systems” approach to behavioral support for smoking cessation in LCS, may act as an important catalyst for behavior change. For example, providing additional cessation support for the wider contextual influences of smoking may aid smoking cessation, and therefore, the use of social support interventions that target stress management and coping skills should be utilized with the target population.^49,50^ Additionally, connecting the target population with volunteer organizations and local community groups has the potential to positively affect health and well-being directly (eg, through lowering stress) and indirectly (eg, by improving access to local services). Embedding social prescribing into behavioral support for smoking cessation in a lung screening setting may assist in improving social isolation and poor mental health.^51^

This study provides an in-depth understanding of the beliefs surrounding smoking and smoking cessation and further potential psychosocial factors that influence those attending LCS. To promote cessation in this population, interventions should aim to encourage positive beliefs about smoking cessation aids as part of a person-centered and supportive approach. It is important that interventions address the context of social isolation and a lack of positive support for smoking cessation that exists in this population. Adopting a holistic approach to behavioral support for smoking cessation in a lung screening setting may act as an important catalyst for behavior change and focus attention on the interconnections between the individual, their community, and other environmental factors that influence motivation to stop smoking.

## Supplementary Material

Supplementary material is available at *Nicotine and Tobacco Research* online.

ntad245_suppl_Supplementary_Appendix

## Data Availability

Data sharing is available upon reasonable request. Please contact the corresponding author.
