# Correction to: Barriers and Facilitators to Engaging in Smoking Cessation Support Among Lung Screening Participants

**DOI:** 10.1093/ntr/ntae034

**Published:** 2024-02-28

This is a correction to: Pamela Smith, Harriet Quinn-Scoggins, Rachael L Murray, Grace McCutchan, Annmarie Nelson, Graham Moore, Matthew Callister, Hoang Tong, Kate Brain, Barriers and Facilitators to Engaging in Smoking Cessation Support Among Lung Screening Participants, Nicotine & Tobacco Research, 2023;, ntad245, https://doi.org/10.1093/ntr/ntad245

The originally published version of this manuscript has been updated to correct author Matthew Callister’s name and affiliations.

