# Supplementary material for: Barriers and Facilitators to Engaging in Smoking Cessation Support Among Lung Screening Participants
Source: Nicotine Tob Res. 2023 Dec 10;26(7):870–7. doi: 10.1093/ntr/ntad245 (PMC11190054; doi:10.1093/ntr/ntad245)
Supplement: ntad245_suppl_Supplementary_Appendix [file ntad245_suppl_supplementary_appendix.docx]

**Appendix 1 YESS Decliner Interview- Topic guide**

***Rationale****: to gain views on:*

*(a) Reasons why the participant declined to see the SCP for the YESS study and what could be done to encourage engagement with the study*

*(b) Their views on stopping smoking and their current motivation to stop smoking*

*(c) Confidence/ self-efficacy around quitting smoking*

*(d) Their previous quit attempts*

*(f) Impact of their social network on smoking/quitting*

*(g) Any comorbid conditions that may impact their motivation to quit/ previous quit attempts*

Reasons for declining

1. Can you tell me about why you decided:
2. How did you feel being approached about giving up smoking on the van?
3. What did you think of the conversation you had with the smoking cessation practitioner on the van?
   - How did talking about stopping smoking on the van make your feel?
   - Did you feel listened to?
   - Did you feel you were being told what to do?
4. Was there anything that you did not like about your experience on the van?
   - How do you think could this be improved?
5. What, if anything, do you think could have encouraged you to:
   - Part 1 decliner- see the smoking cessation practitioner on the day of your lung health check?
   - Part 2 decliner- continue seeing the smoking cessation practitioner after your lung health check?

**Previous quit attempts/ use of smoking cessation service and NRT**

1. If it is OK with you, I’m now going to ask you some questions about times when you have tried to stop smoking. Have you made a serious attempt to stop smoking before? Could you please tell me about times when you seriously tried to stop smoking? (meaning you decided that you would try to make sure you never smoked again)
   - IF YES- Can you tell me more about your quit attempt(s) i.e. how many times have you made a serious attempt to quit smoking?
   - What is the longest that a quit attempt has lasted for you? Why would you say this/these quit attempts did not last?
   - How have your previous attempts to stop smoking influenced how you think about stopping smoking now?
   - **What do you think would have helped you to be successful during your previous quit attempt/attempts?**
     1. **If something would have motivated you to continue not smoking, what do you think it would have been?**
     2. **Did you ever feel you needed more to help you quit when you previously tried? What do you think would have helped?**
   - Have you ever used nicotine replacement products in the past?
     1. IF YES- how did you find using nicotine replacement products?
   - Have you ever used an e-cigarette?
     1. IF YES-What did you like about using an e-cigarette? What did you not like about using an e-cigarette?
   - Have you ever been to behavioural counselling to help you quit smoking (i.e. a local stop smoking service group, community versus primary care support)?
     1. IF YES- What did you think about it? What did you like about the behavioural support? Was there anything you didn’t like about the support? If so, what did you not like?
   - What do you know about local Stop Smoking Services in your community?
     1. Have you ever used them? Would you ever consider using them if you decided to quit smoking?
2. What would you like to see in your community that may help you make an attempt to stop smoking?
   - How do you think your local stop smoking services could help you make an attempt to stop smoking?
   - What support would like to have available to you if you decided to stop smoking?

**Motivation to Stop Smoking/ Quitting self-efficacy**

1. **Facilitate the participant to complete the Motivation to Stop Smoking Scale (Appendix 1) and use this to elicit discussion around motivation to quit:**
   - IF 1- Why do you not want to stop smoking?
   - IF 2- Why do you not want to stop smoking?
   - IF 3- Why do you want to stop smoking? Why do you think you haven’t thought about when you would like to stop smoking?
   - IF 4- Why do you really want to stop smoking? Why do you think you’re unsure of when you will quit?
   - If 5- Why do you want to quit smoking?
   - If 6 or 7- Why do you really want to quit smoking?
   - If 8- Why do you think you are unsure about whether you want to quit smoking?
2. **How do you feel about stopping smoking?**
   - What would make it difficult for you to quit smoking?
   - What would make it difficult for you to stop smoking?
   - What impact do you think stopping smoking could have on your health?
   - Can you think of any benefits to you personally if you gave up smoking?
   - Can you think of any negatives if you gave up smoking?
   - What (if anything) would make you attempt to stop smoking?
3. **How important is stopping smoking to you?**
4. **How confident are you that you could quit smoking?**
5. **What do you think could increase your motivation to stop smoking?**
   1. **Is there anything that could assist you in feeling more motivated to quit smoking?**

**Social networks and their impact on smoking/ quitting smoking**

1. ***During rapport building gauge whether the participant have family/friends***

**Smoking and social influences**- I’m interested to know about smokers in your social circle. Do many of the people who are closest to you i.e. your family and friends smoke? (if they do not then re-frame to explore how they feel the lack of social support impacts their smoking behaviour)

- - How do you feel this affects your smoking? Could you tell me a bit about how often you smoke with those closest to you? If they told you that they had stopped smoking, how would that make you feel/ what would your reaction be? How do you think they would feel if you quit smoking and/or what would they say if you wanted to stop smoking?
  - How supported by your family and friends would you feel if you decided to quit smoking? [if a previous quit attempt has been made] How much support did your family and friends provide during a quit attempt?
  - Do any of your friends/family use e-cigarettes? What would your friends/family think of you using an e-cigarette?
  - Tell me about any social situations (or experiences) that make quitting difficult? Or any situations that have helped you to make a quit attempt?
  - *Tailor this according to what (if any) close social network they describe earlier in the interview-*How do you feel your close social network/community affects your smoking behaviour?

1. If you could have any support to help you stop smoking or feel more motivated to stop smoking, what would it be?
   - Why do you feel this form of support would help you?
   - How do you think family/friends, members of the community or NHS could help you quit smoking?
   - How do you think emotional support would help you? i.e. congratulating you on giving up
   - How do you think physical support would help you? i.e. not smoking around you
2. How easily available/accessible do you feel stop smoking services in your local area are?
   - What do you think could be done to improve stop smoking services?

**Health conditions that may impact quitting smoking**

1. **Do you have any health conditions? If so how do you feel these impact you and your smoking habits?**
   - How has this issue affected your previous quit attempts?
   - How does smoking impact your pre-existing health issue?
